# Overexpression of *Liriodendron tulipifera JAG* Gene (*LtuJAG*) Changes Leaf Shapes in Transgenic *Arabidopsis thaliana*

**DOI:** 10.3390/ijms23031322

**Published:** 2022-01-25

**Authors:** Lingmin Wei, Shaoying Wen, Zhonghua Tu, Yanqing Zhao, Huogen Li

**Affiliations:** 1 Key Laboratory of Forest Genetics & Biotechnology of Ministry of Education, Co-Innovation Center for Sustainable Forestry in Southern China, Nanjing Forestry University, Nanjing 210037, China; lmwei2019@njfu.edu.cn (L.W.); Winnie@njfu.edu.cn (S.W.); zhonghuatu@njfu.edu.cn (Z.T.); zhaoyanqing@njfu.edu.cn (Y.Z.); 2 College of Forestry, Nanjing Forestry University, Nanjing 210037, China

**Keywords:** *Liriodendron tulipifera*, *LtuJAG*, leaf polarity, transformation, *Arabidopsis thaliana*

## Abstract

In *Arabidopsis thaliana*, *JAGGED* (*JAG*) is a transcription inhibitor that controls the development of leaf polarity and regulates the expression of genes controlling lateral organ formation. *Liriodendron tulipifera* is an ornamental tree with extraordinary tulip-shaped flowers and goose web-like leaves, this is one of the suitable plants for morphological development research. To investigate the potential functions of the *LtuJAG* gene, we isolated the full-length *LtuJAG* from *L. tulipifera*, transferred it into *A. thaliana* via agrobacterium-mediated transformation, and monitored its expression pattern. Subcellular localization showed that *LtuJAG* was located in the nucleus. RT-qPCR assays indicated that *LtuJAG* was expressed mainly in leaf buds and flowers, but not in mature leaves and stems. GUS staining results showed that *LtuJAG* was expressed in the shoot apical meristem (SAM). Overexpressing *LtuJAG* changed *A. thaliana* leaf shapes, causing a moderate serration and a slight asymmetric distribution in the medio-lateral and proximal-distal axes. Ectopic expression of *LtuJAG* induced the expression of lateral organ boundary suppressors *JAGGED LATERAL ORGANS* (*JLO*) and *ARABIDOPSIS THALIANA HOMEOBOX1* (*ATH1*). It also repressed the expression of the apical meristem suppressor class-1 *KNOX* gene (*KNOX I*) and altered endogenous hormone levels. Our results suggest that *LtuJAG* plays a role in negatively regulating leaf polarity formation in *L. tulipifera*.

## 1. Introduction

The organ development of seed plants usually undergoes two opposite modes: (1) root and stem development, which displays overall radial symmetry; (2) development of lateral organs such as leaves and flowers, which show an obvious asymmetry [1]. The leaves of seed plants evolved from lateral branches, initiating from the peripheral zone (PZ) of the shoot apical meristem (SAM) and developing into flattened structures [2]. Genetic evidence has revealed that leaves underwent four morphological changes during their evolution: curling into a cone, developing short and flexible petioles as well as serrated edges, forming compound pinnate leaves, and stacking with each other along the branches to adjust the phyllotaxis and reduce drag [3]. Due to their morphological plasticity, leaves are ideal research subjects for under-standing organ morphogenesis and environmental adaptability.

**Figure 1:**
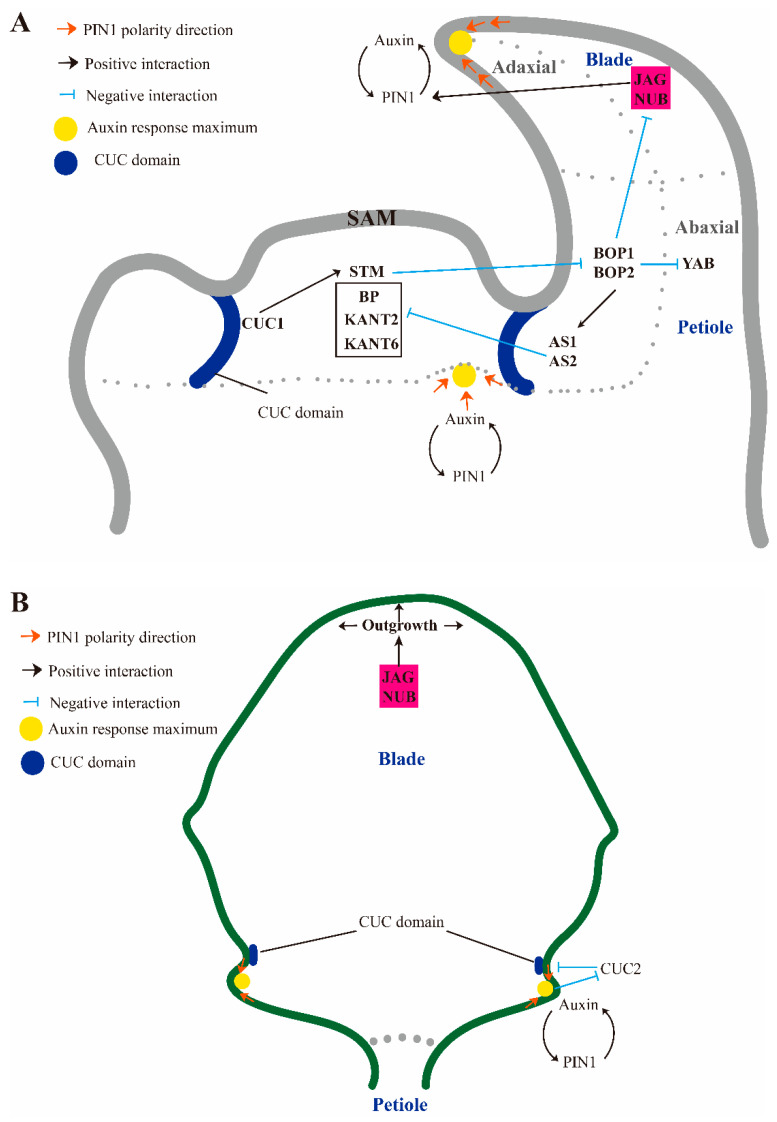
Regulation pattern of leaf margin morphogenesis in *A*. *thaliana*. (**A**) Regulation network controlling leaf polarity. BOP_S_ in the petiole directly activate *AS1/2* to repress BP and exclude JAG-like factor from the petiole and restrict to the leaf margin thereby promoting leaf polarity differentiation; (**B**) Regulation network of leaf margin serration induced by boundary gene. JAG promotes PIN1 convergence at the tip of serrations along leaf margin and interacts with boundary genes *CUC2*.

The development of the leaves includes three processes: leaf initiation, leaf polarity establishment, and leaf morphology modulation [4]. Leaves emerge from a mass of stem cells at the SAM via lateral differentiation, thereby forming leaf primordia. The stem cells differentiate and proliferate along the asymmetric polarity of the adaxial-abaxial, medio-lateral, and proximal-distal axes to establish leaf polarity [5]. After the establishment of leaf polarity, its shape is formed by continues cell division and expansion. Many genes involved in leaf development have been identified, including *KNOX I* family genes, *ASYMMETRIC LEAVES1* (*AS1*)/*ROUGH SHEATH2* (*RS2*)/*PHANTASTICA* (*ARP*), *PIN-FORMED1* (*PIN1*), *BLADE-ON-PETIOLE 1/2* (*BOP1/2*), and *CUP-SHAPED COTYLEDON* (*CUC*) (Figure 1). The *KNOX I* family genes including *SHOOT MERISTEMLESS* (*STM*), *BREVIPEDICELLUS* (*BP* or *KNAT1*), *KNOTTED IN ARABIDOPSIS THALIANA 2* (*KNAT2*), and *KNAT6*, which are essential for maintaining SAM activity, and their downregulation are required for leaf initiation [6,7,8]. This downregulation is contributed by ARP transcription factors. The *AS1* and *AS2* genes form a complex with the regulatory motifs CWGTTD (located on the *BP* promoter) and KMKTTGAHW (located on the *KNAT2* promoters) [9]. Finally, they performed negative regulation of *BP* and *KNAT2* together [10]. *BOP1* and *BOP2* can also repress *BP* by directly promoting the expression of *AS2* [11,12] (Figure 1A). Moreover, *BOP1* and *BOP2* participate in the establishment of adaxial-abaxial polarity through *AS2* (adaxial fate regulator) activation and *YABBY* (*YAB*, abaxial fate regulator) repression [13] (Figure 1A). *BOP1* and *BOP2* proteins also function redundantly with *AS1* and *AS2* in the establishment of proximal-distal polarity [11] (Figure 1A). Notably, the PIN1–CUC regulation pathway plays important roles in leaf development—establishment of organ boundary and determining the location of serration during leaf morphology modulation [14] (Figure 1A,B). Moreover, SAWTOOTH 1 (SAW1) and SAW2 transcription factors also participate in leaf serration formation. The double mutations of *SAW1* and *SAW2* initiate the ectopic expression of *BP* at the leaf margin, resulting in the lobed leaf and indicating that *SAW1* and *SAW2* are involved in the development of leaf margins by negatively regulating the expression of *KNOX I* [15]. While the molecular development model from leaf primordia to maturity discussed above is generally accepted, the exact process of leaf morphogenesis is still unclear.

In recent years, some boundary genes have been found to be involved in the regulation of organ polarity. *JLO* has been shown to activate the expression of *STM* and *BP* and inhibit the expression of the auxin efflux transporter *PIN1* [16], and *JAG* and its paralog *NUBBIN* (*NUB*) are known to be negatively regulated by *BOP* in lateral organs [17,18] (Figure 1A). Further studies found that *JAG* positively regulates the expression of *PIN1* in tomato leaves [19] (Figure 1B). Loss of function mutations at *JAG* sites leads to abnormal lateral organs in *A. thaliana*, including narrow leaves and serrated petals [20,21]. Research has also shown that *JAG* controls the coordination of the cell cycle and growth in calyx primordia [22]. In addition, *JAG* controls not only the growth rate of cells but also the polarized growth of organs. *JAG* and *AS1*/*AS2* repress the expression of boundary-specific genes in sepals and petals to maintain normal organ initiation within the boundary zone [23]. Many studies have confirmed the regulatory role of *JAG* in petal growth and development, but the molecular mechanism of *JAG*-mediated leaf morphogenesis is not clear. We set out to explore whether a better understanding of the expression pattern of *LtuJAG* may be helpful in improving the desired aesthetic qualities of leaves, thereby changing the utilization rate of light and other energy and assisting selective breeding programs for the improvement of ornamental tree species.

## 2. Results

### 2.1. Cloning and Sequence Analysis of LtuJAG

**Figure 2:**
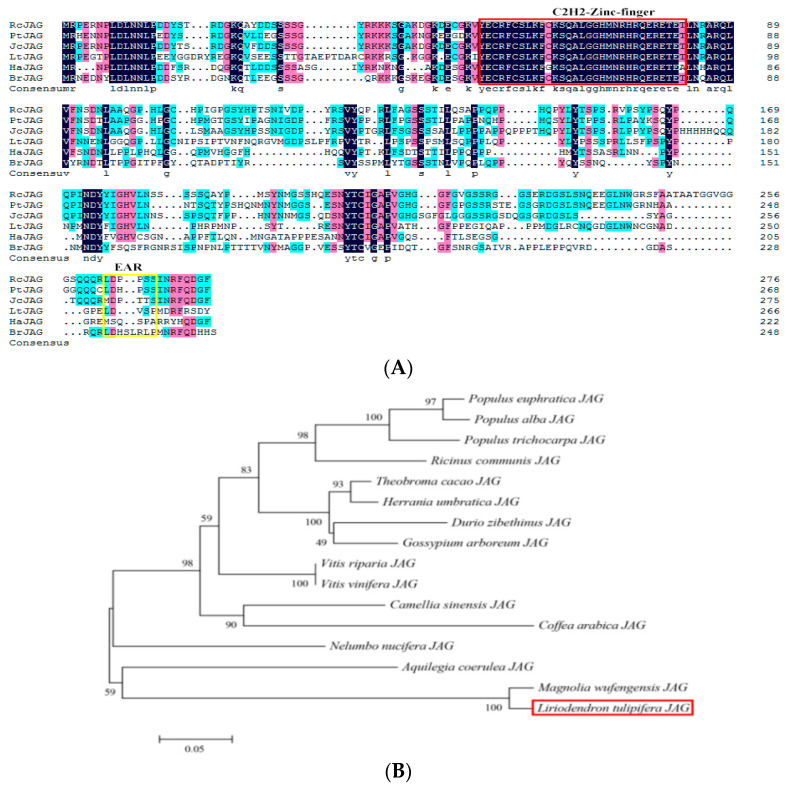
Sequence and phylogenetic analyses of LtuJAG protein. (**A**) The multiple sequence alignment of LtJAG protein with JAG-like protein in *Ricinus communis* (RcJAG, XP_025015302.1); *Populus trichocarpa* (PtJAG, XP_024466075.1); *Jatropha curcas* (JcJAG, XP_020538088.1); *Helianthus annuus* (HaJAG, XP_021975637.1); *Brassica rapa* (BrJAG, XP_009105303.1). The highly conserved core sequence Homeodomain is represented by a red box. The EAR motif is indicated by a yellow box; (**B**) Phylogenetic relationships among JAG proteins from different plant species. LtuJAG is indicated by the red box.

Full-length *LtuJAG* (1344 bp; ORF, 801 bp) encoded a protein comprising 267 amino acid residues with a molecular weight of 29.50 kDa and a theoretical isoelectric point (pI) of 8.05. The hydrophilicity of the protein was −0.856 with an instability coefficient of 58.05, indicating that it was likely to be unstable and hydrophobic. The amino acid sequence of *LtuJAG* was used as a query to perform a BLASTP search to explore its structural conservation and phylogenetic position. Thirteen homologous protein sequences from other plant species showed that the *N*-terminus comprising the C_2_H_2_ zinc-finger domain was conserved while the EAR motif was not, indicating that *LtuJAG* belongs to the C_2_H_2_ zinc-finger family (Figure 2A). The alignment of these sequences showed a high degree of conservation relative to *LtuJAG*, varying from 91.35% (*Magnolia wufengensis*, QAY29215.1) to 51.05% (*Populus euphratica*, XP_011007835.1). The phylogenetic tree showed that JAG proteins from *L. tulipifera* and *M. wufengensis* were clustered together (Figure 2B).

### 2.2. Subcellular Localization of LtuJAG

**Figure 3:**
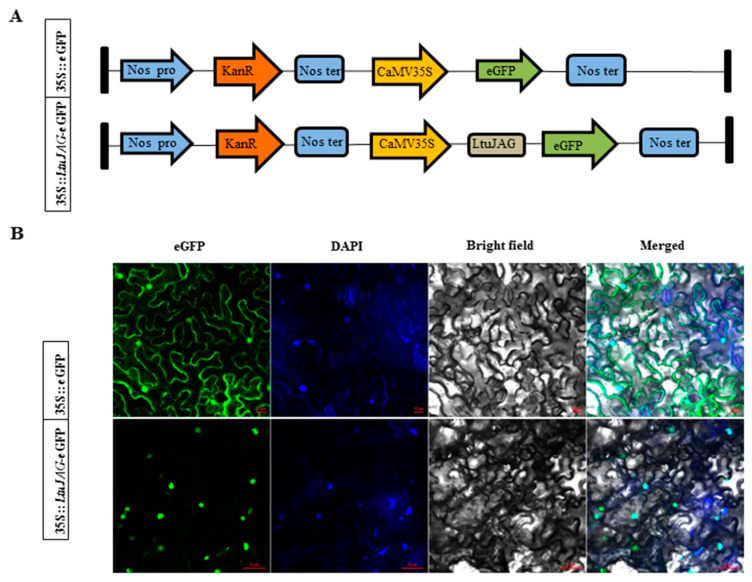
Subcellular localization of LtuJAG protein. (**A**) The construct of 35S::LtuJAG-eGFP and 35S::eGFP (eGFP: green fluorescent protein; NOS: nopaline synthase gene; KanR: Kanamycin resistance gene); (**B**) Subcellular localization of the 35S::LtuJAG-eGFP protein in tobacco epidermal cells. At 2 days after transfection, eGFP and DAPI (4′,6′-diamidino-2-phenylindole, and nucleus specific dye) signal was observed by confocal fluorescence microscopy, 35S::eGFP was used as a control. Scale bars = 20 and 50 µm.

Subcellular localization using LOCALIZER (http://localizer.csiro.au/) (accessed on 2 July 2020) [24] predicted that *LtuJAG* would be found in the nucleus. Green fluorescent protein (GFP) signals of the 35S::eGFP construct were inserted into the nucleus and cell membrane, while the signal of LtuJAG::eGFP was only localized in the nucleus of *N. benthamiana* leaves (Figure 3B). DAPI (4′,6′-diamidino-2-phenylindole) dye was utilized to stain the nucleus to stimulate a fluorescent blue signal. The resulting fluorescent cyan signal further confirmed that LtuJAG was located in the nucleus, which is consistent with transcription factor patterns (Figure 3B).

### 2.3. Tissue Expression Pattern of LtuJAG

**Figure 4:**
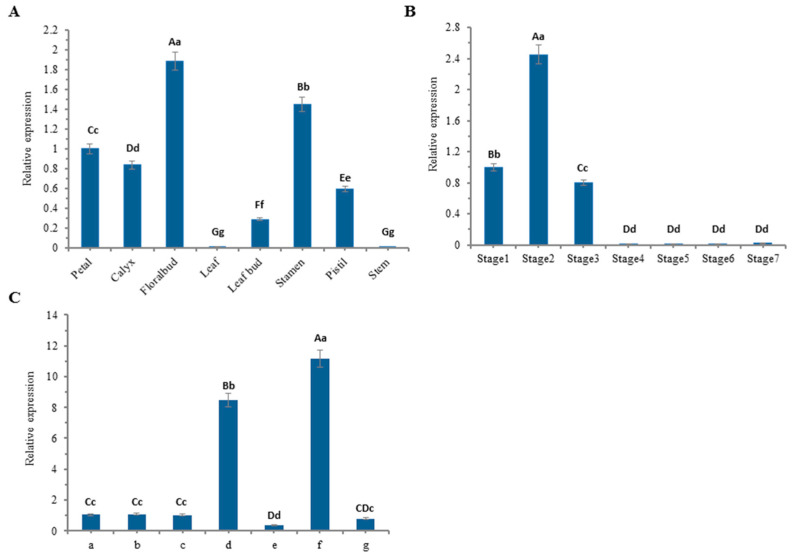
Quantitative RT-PCR analysis of transcript levels of *LtuJAG* in *L. tulipifera*. *LcACTIN97* gene was used as an internal control to normalize the values of target transcripts. (**A**) Transcript levels of *LtuJAG* in different tissues; (**B**) Transcript levels of *LtuJAG* at different stages of leaf development; Stage 1–2: leaf bud growth stage; Stage 3: young leaf stage; Stage 4–6: mature stage; Stage 7: senescence stage; (**C**) Transcript levels of *LtuJAG* at different parts of leaf in *L. tulipifera*, a, c and e: leaf tooth tip, b and d: leaf tooth sinus, g: middle of leaf, f: petiole. Date represented the means (±SD) from the three replicates, and the error bars represent the standard deviation between the replicates. Data were analyzed statistically using the one-way ANOVA Tukey’s test. Different letters indicate significant differences at *p* < 0.01 and *p* < 0.05 level.

To verify the expression pattern of *LtuJAG* in different tissues of *L. tulipifera*, we examined the mRNA of *LtuJAG* in eight *L. tulipifera* tissues (Figure 4A). Expression in flower buds was significantly higher than that of the other tissues, followed by expression in the stamen, petals, calyx and leaf buds, with stems and leaves showing the not expression. This suggests *LtuJAG* may play an important role in the development of flower organs.

The time-specific expression pattern of *LtuJAG* during leaf development determined by sampling the leaf bud growth stage (stages 1 and 2), young leaf stage (stage 3), mature stage (stages 4 to 6), and senescence stage (stage 7) showed that *LtuJAG* expression increased with the gradual expansion of leaf buds at the bud growth stage (Figure 4B). The expression of *LtuJAG* decreased with the growth of leaves at the leaf-spreading stage. *LtuJAG* was not expressed during leaf maturation and senescence, indicating that it may be expressed during vigorous meristem growth of *L. tulipifera* but does not participate in the regulation of leaf growth and development at maturity.

To further confirm the leaf space-specific expression pattern of *LtuJAG*, we divided the leaf margin of *L. tulipifera* into tooth tips and sinuses. *LtuJAG* expression was markedly higher in the petiole of the leaf than in the other parts, and it was generally higher in leaf tooth sinuses (d) than in leaf tooth tips (a, c, and e) (Figure 4C). These results suggest that *LtuJAG* may play a direct regulatory role in leaf margin development as well.

### 2.4. LtuJAG Promoter Is Leaf-Specific in Transgenic A. thaliana

To detect tissue specificity, the *LtuJAG* promoter fragment (named *pLtuJAG*) located approximately 2Kb upstream of the *JAG* gene in *L. tulipifera*, was cloned, sequenced and analyzed for potential regulatory elements (Table S2), including light-responsive cis-elements, such as Box 4, G-box and I-box, as well as TCCC-motifs. The abiotic stress response elements included LTR (low temperature), MYC (cold), STRE (thermal induction), TC-rich repeats (defense and stress responsiveness), and WRE3 (damage). Furthermore, a few hormone-responsive elements were identified, such as ABRE (ABA), AuxRE (Auxin), AuxRR-core (Auxin), ERE (ethylene), TATC-box (GA), and TCA-element (salicylic acid). Sequence analysis of the *LtuJAG* promoter region implies that the *LtuJAG* gene may be implicated in regulating a range of processes, including responses to light signals, abiotic stresses, and hormones.

**Figure 5:**
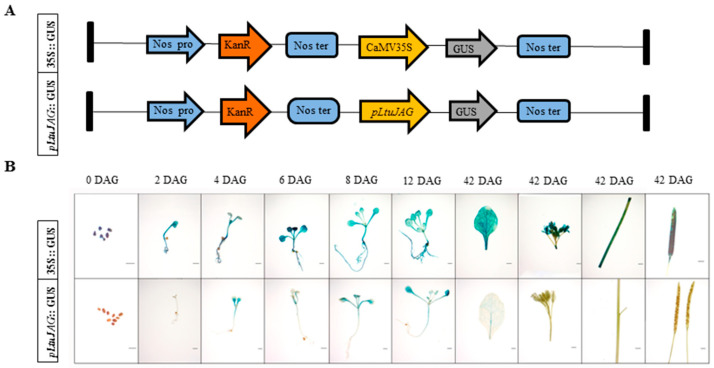
GUS histochemical assays in transgenic *Arabidopsis* T_2_ overexpression lines. (**A**) The T-DNA construction diagram used for *Arabidopsis* transformation. GUS: β-glucuronidase gene; Nos: nopaline synthase gene; KanR: Kanamycin resistance gene; (**B**) Histochemical staining in seedlings (two-leaf stage, four-leaf stage and six-leaf stage), rosette leaf, stem, pods, inflorescences, and seeds sampled during the vegetative and reproductive stage form transgenic T_2_ *Arabidopsis* seedlings harboring constructs with GUS expression driven by the CaMV 35S promoter (35S::GUS) and *pLtuJAG*. Photographs were taken 2 days, 4days, 6 days, 8 days, 12 days, and 42 days after seed germination. DAG: day after germination. Scale bar = 1 mm.

To confirm the core elements responsible for meristem development, the *pLtuJAG* sequence was fused to the GUS reporter gene (pLtuJAG::GUS) and transformed into *A. thaliana*, pLtuJAG-induced GUS expression was observed during the two-leaf, four-leaf, six-leaf and flowering stages of T_2_ overexpression lines using histochemical staining (Figure 5A,B). After germination for 2 days, GUS activity of seeds and seedlings was not expressed. At the stage of leaf bud differentiation (4–12 days), GUS expression was not detected in the radicle. Concurrently, the *LtuJAG* promoter drove GUS to be stably expressed in leaves and hypocotyls. With the growth of seedlings, GUS activity in leaf buds gradually decreased, while gradually increasing in newly differentiated leaves (Figure 5B). It was only expressed in leaves, but not in inflorescences, pods, and stems at the reproductive stage (Figure 5B). Furthermore, we observed that the promoter activity of *LtuJAG* was the strongest in the main leaf vein, followed by the secondary leaf vein, and weak GUS expression was detected in mesophyll cells, indicating that the *LtuJAG* promoter is exclusively expressed in leaf organs. From these observations, it can be inferred that *pLtuJAG* is a leaf bud meristem-specific promoter.

### 2.5. Phenotype of Transgenic A. thaliana

**Figure 6:**
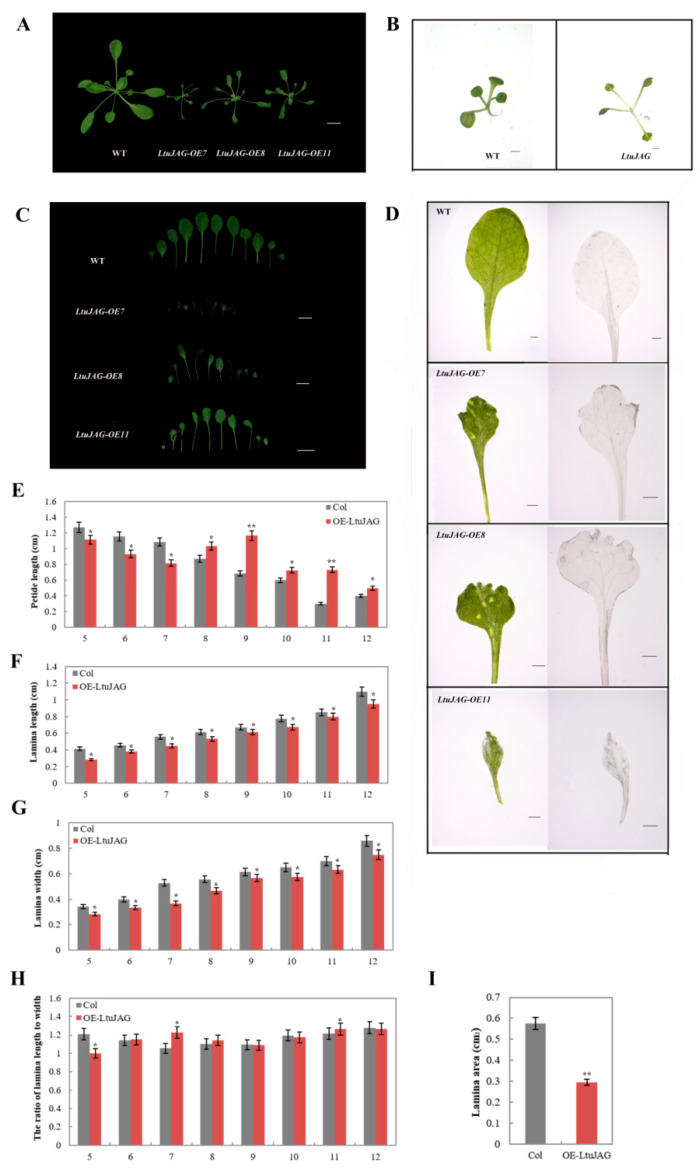
The margin serration phenotype of transgenic *LtuJAG* lines. (**A**–**D**): The transgenic line 35S::LtuJAG exhibited a phenotype causing petiole elongation, leaf serration and small leaf area at the 10 days (**B**) and 32 days (**A**,**C**,**D**) after seed germination. Scale bar = 1 mm and 1 cm; (**H**,**I**): Quantification of leaf lobes of transgenic *LtuJAG* lines, Col-0 was used as a wild-type control; (**E**): Quantification of the petiole lengths of 5th leaves to 12th leaves; (**F**): Quantification of the lamina length of 5th leaves to 12th leaves; (**G**): Quantification of the lamina width of 5th leaves to 12th leaves; (**H**): Quantification of the ratio of the lamina length to width; (**I**): Quantification of lamina area of 5th leaves. Leaves of plants grown for 32 days were examined, error bars showed SD (n ≥ 12). The asterisks indicate significant difference from the WT using the Student’s *t*-test (** *p* < 0.01, * *p* < 0.05).

The coding sequence of *LtuJAG* was fused to the 35S promoter (35S::LtuJAG) and transferred into *A. thaliana*. Approximately 32 T_1_ generation positive plants were produced. Seven of these overexpression lines had the phenotype of small leaf area (*LtuJAG*-OE11), including two with a phenotype producing serration, *LtuJAG*-OE7 and *LtuJAG*-OE8 (Figure 6A,C). The transgenic line 35S::LtuJAG-OE7 exhibited a phenotype causing small leaf area, we also measured lamina length, width, and area in wild type and overexpression lines. Compared with the wild type, the petiole of the overexpression strain became longer (Figure 6E), the lamina length, width, and area were reduced (Figure 6F,G,I) The ratio of lamina length to width remained unchanged (Figure 6I), indicating that the overexpression line 35S::LtuJAG-OE7 altered leaf size by simultaneously reducing the rate of leaf cell expansion in both the medio-lateral and proximal-distal axis directions. A range of defects was observed, including irregular curls and lobes at the distal ends of the leaves (Figure 6D), thinner mesophyll tissue in the lobed part of the leaves, abnormal vein growth, and narrowing of the proximal ends forming a wedge. This latter also induced some petiole elongation, and only petioles remained in some severely defective leaves. This phenotypic change in the absence of leaf cells suggests suppression of leaf polarity had occurred.

The transgenic line 35S::LtuJAG-OE8 exhibited a phenotype causing petiole elongation and leaf serration at the four-leaf stage (Figure 6B) while showing dorsal leaf margin curling at the mature stage (Figure 6A,C). This was caused by accelerated proliferation and expansion of mesophyll cells on the adaxial blade, while the cell division speed on the abaxial blade remained unchanged. To a greater extent, the leaves showed a moderate serration and slight asymmetric distribution along the medio-lateral axis, which was consistent with the heteromorphic leaves of a mutant [20].

### 2.6. Expression of Genes Related to Leaf Development in Transgenic A. thaliana

**Figure 7:**
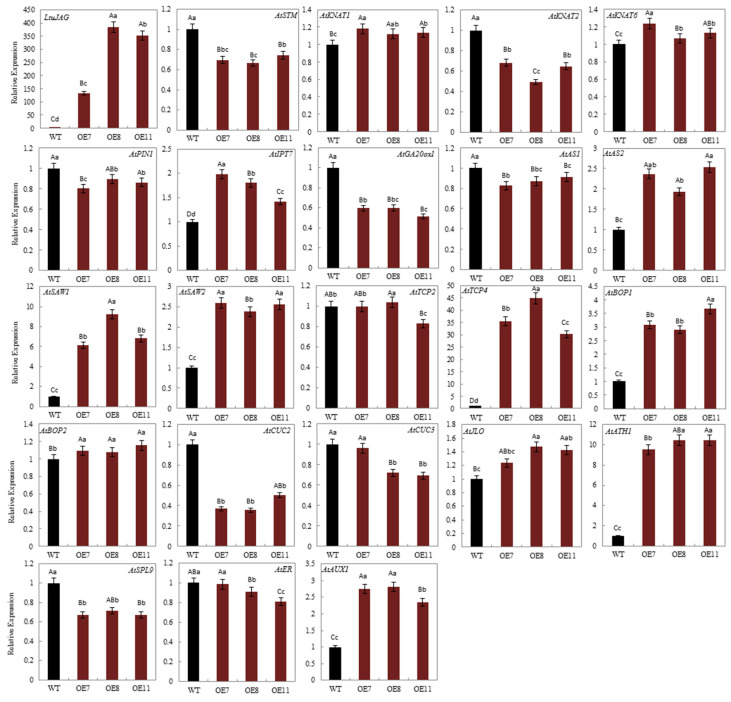
Quantitative RT-PCR analysis of *LtuJAG* and genes in relation to leaf development in the ten-day-old wild type (WT) and three independent *LtuJAG* overexpression lines, OE7, OE8 and OE11. *ACTIN2* served as the reference gene. The value of genes expression level in WT was set to “1” as a control, the error bars represent the standard deviation among three biological replicates. Data were analyzed statistically using the one-way ANOVA Tukey’s test. Different letters indicate significant differences at *p* < 0.01 and *p* < 0.05 level.

Since the formation of leaf morphology occurs during the transition from leaf primordia initiation to polarity establishment, we selected 22 genes that have been reported to play a part in this process. The heterologous overexpression of *LtuJAG* resulted in a significant increase in the expression of *LtuJAG* in overexpression lines (OE7, OE8, and OE11), indicating that we successfully identified the overexpression lines of T_3_ generation (Figure 7). Compared with the wild type, 35S::LtuJAG increased the expression of *IPT7* and decreased the expression of *GA20ox1* at the leaf primordia, suggesting that *JAG* promotes antagonism between *GA20ox1* and *IPT7*, thus maintaining the dynamic balance of hormones during development. 35S::LtuJAG downregulated the expression of *STM*, *KNAT2*, *SBP-LIKE9* (*SPL9*), and *CUC2/3*, and upregulated the expression of *AS2*, *JLO*, *BOP1*, *SAW1/2*, *ATH1*, *AUX1* and *TEOSINETE BRANCHED1/CYCLOIDEA/PROLIFERATING CELL FACTOR 4* (*TCP4*) at the leaf primordia. The expressions of *KNAT1*, *KNAT6*, *PIN1*, *AS1*, *BOP2*, *TCP2*, and *ER* were not significantly different between WT and the overexpression lines (Figure 7). Therefore, we speculate that *JAG* may participate in leaf primordia differentiation and leaf polarity formation through multiple regulatory pathways, but determining by which regulatory pathways would require further investigation.

## 3. Discussion

### 3.1. Domain Function in LtuJAG

A 1344 bp cDNA full-length sequence named *LtuJAG* encoding 267 amino acid proteins was identified within the leaf bud cDNA of *L. tulipifera*. Sequence analysis of *LtuJAG* showed that *LtuJAG* had similar conserved domains with *JAG* in *Ricinus communis*, *Populus trichocarpa*, *Jatropha curcas*, *Helianthus annuus*, and *Brassica rapa*. A single 31 amino acid C_2_H_2_ type Zinc- finger motif (amino acid position 60–91), which may have a role in DNA binding, and a short nine amino acid ERF-associated amphiphilic expression (EAR) motif (amino acid positions 250–258) at the amino acid end were observed to function as repressors. These results demonstrate that JAG belongs to the C_2_H_2_ type zinc-finger transcription factor family. Homology analysis showed that the amino acid sequence of LtuJAG was 51.05–91.35% similar to that of other species, especially the woody plant *M. wufengensis*. *LtuJAG* is predicted to function similarly to *AtJAG*, a class of transcriptional inhibitors that regulate processes involved in organ morphological development and polar growth [25].

Promoters are central to processes of transcriptional regulation and fundamental to the study of gene expression and regulation. Analysis of cis-acting elements showed that their most common products were hormones, such as aux (AuxRE), GA (TATC-box), abscisic acid (ABRE), salicylic acid (TCA-element), and ethylene (ERE). The functions of auxin and GA are associated with the developmental function of *JAG* in regulating the three-dimensional morphology of plant organs. Moreover, plants synthesize abscisic acid to initiate defense mechanisms against stress [26]. Salicylic acid and ethylene are endogenous signaling molecules that activate plant hypersensitivity and systemic acquired resistance [27,28]. MYB and WRKY are involved in regulating the dual response of plants to biotic and abiotic stresses [29,30]. Therefore, we speculate that the *LtuJAG* gene is a transcription factor that participates in regulating development and resistance to stress.

### 3.2. Expression Pattern of LtuJAG during Leaf Shape Formation

Our study showed that LtuJAG was located in the nucleus of tobacco, while by observing AtJAG::GFP fusion protein under control of *AP1* promoter in the epidermal cells of pedicels of flower primordia, AtJAG was located in the nucleus. This suggests that JAG has the characteristics of a typical transcription factor [21]. *LtuJAG* was specifically expressed in flower organs, such as flower buds, petals, and stamens. High concentrations of *JAG* RNA in rice have been detected in the inflorescence meristem, axial meristem, flower organ primordia, stamen, pistil, anther, and integument [31,32,33], demonstrating that *JAG* has a certain impact on the development of each floral organ. *LtuJAG* was only expressed in leaf buds at the vegetative stage, and not in mature leaves or stems, which is consistent with other observations of *A. thaliana* by Dinneny et al. [21]. To gain further insight into the expression pattern of *LtuJAG*, we observed that the *LtuJAG* promoter was stably expressed in leaves and hypocotyls at the vegetative stage of Arabidopsis, and its expression in the leaves gradually increased over time. *AtJAG* promoter-driven *GUS* gene expression was highest in the radicles and hypocotyls 12 h after germination, gradually decreased in the cotyledons and hypocotyls at 3 and 4 days, and increased in the radicles [34], which further explains the specific expression of *JAG* at the early stage of apical meristem differentiation. In conclusion, we demonstrated that *JAG* is a transcription factor that regulates leaf and floral organ development.

### 3.3. Overexpression of LtuJAG Results in Leaf Polarity Defect

The adaxial axes of leaf primordia face towards the meristem while abaxial axes face away from it. The formation of the adaxial-abaxial axis is fundamental for establishing the medio-lateral axis and proximal-distal axis of the leaf [1]. Overexpression of *LtuJAG* in *A. thaliana* resulted in the curling of adaxial tissue towards the abaxial axis. Cytological observations indicate that defects within the thick-walled cells on the abaxial axis of leaves are responsible for curling [35], indicating that *LtuJAG* affects cell development. Intriguingly, we found that *LtuJAG* was ectopically expressed in leaves with deep serrations at the tip and margin. Study of *KIP-RELATED PROTEIN2* (*KRP2*) revealed that the appearance of deep serration was caused by a proliferation defect in total cells, and the degree of lobe formation was related to the number of cells [36]. Moreover, the *SHALLOT-LIKE1* (*SLL1*) gene in rice controls the development of leaf adaxial thick-walled cells by regulating programmed cell death (PCD) to establish leaf adaxial polarity [35]. Thus, the rate of cell division and proliferation can directly affect the final morphology of an organ, but the mechanisms by which cell division is controlled are not well understood. On the other hand, the establishment of adaxial-abaxial, proximal-distal, and medio-lateral axis polarities appears to be coordinated rather than independent of each other. How the development of these three polarities is coordinated remains to be discovered.

### 3.4. LtuJAG Overexpression Alters the Expression of Genes Related to Leaf Morphogenesis

Numerous studies have confirmed the inhibitory effect of *JAG* on the development of petal polarity, and that petals are derivatives of the apical meristem and homologues of leaves [20,21,22,23]. Therefore, we speculated that *JAG* has an effect on the development of leaf polarity. The mutation of *JAG* in *Arabidopsis* resulted in edge serration of leaves and petals [21]. The overexpression of *JAG* promoted the fusion of cotyledons to varying degrees and had no effect on the growth of mature leaves [20]. We confirmed that *LtuJAG* influenced the establishment of three polar axes of leaves, and further examined the expression of related genes. We observed that *LtuJAG* significantly affected other developmental genes at the differentiation stage of leaf primordia. We further confirmed that *LtuJAG* significantly downregulated the expression of the *KNOX I* gene, *STM*, and *KNAT2*, but had little effect on *KNAT1* and *KNAT6*. This expression pattern is also consistent with the finding that *KNAT1* and *KNAT6* do not participate in the regulation of early morphogenesis in leaf primordia [7,8]. Previous studies have confirmed that *AS1/2*, *BOP1/2*, and *SAW1/2* act as negative regulators of *KNOX I* in the development of leaf primordia [9,11]. Our study found that *LtuJAG* can upregulate the expression of the *KNOX I* negative regulator, indicating that *JAG* may indirectly downregulate the expression of *KNOX I* during the development of lateral meristematic tissue. *LtuJAG* also accelerated the expression of the meristem boundary inhibitor genes *ATH1* and *JLO*. *ATH1* induces the expression of *KNOX I* downstream genes by forming heterodimers with *STM* and BP [37]. Similarly, *JLO* can activate the expression of *STM* and *BP* [16]. In conclusion, we speculate that *JAG* may synergize or promote *KNOX I* upstream activators in leaf primordia differentiation and participate in the positive regulation of *KNOX I* upstream inhibitors.

Other boundary inhibitors, such as *CUC1/2/3*, have been found to be activated by *STM* during the development of apical meristem, and *CUC2* is regulated by *STM* and restricted to the SAM boundary region [38]. The inactivation of *TCP* function contributes to the excessive proliferation of leaf margins, resulting in leaf expansion and curled or serrated leaflets [39,40,41]. Regulation of leaf margin development by *TCP* transcription factors is accomplished by the negative regulation of *CUC1/2/3*. *TCP4* forms dimers with *CUC2* and *CUC3*, respectively, inhibiting the expression of *CUC2/3* [42]. *SPL9* competitively binds *TCP* to form dimers with *CUC* [42], and it is likely that the expression of *SPL9* also affects the expression of *CUC*. This study found that *LtuJAG* can increase the expression of *TCP4* and downregulate the expression of *SPL9* and *CUC2/3*, indicating that *JAG* can also participate in the *TCP4-CUC2/3* regulation pathway, but whether it is relative by *JAG* regulating *SPL9* expression with this pathway requires further investigation.

**Figure 8:**
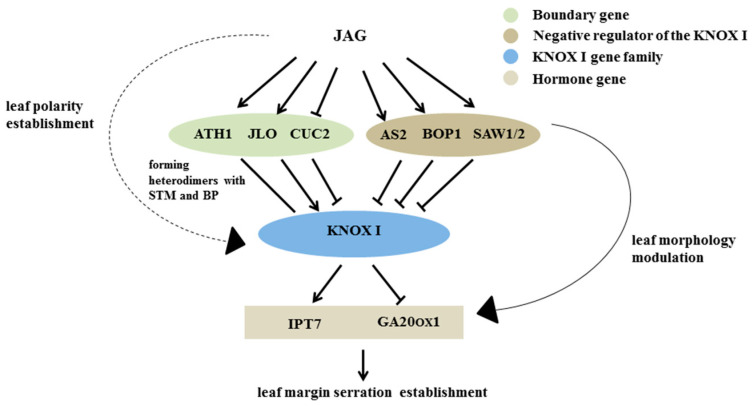
Proposed model for the regulatory mechanism of *JAG*. KNOX I family members are differentially expressed during different developmental stages of plants. The initiation and polarity establishment of leaf organs need to trigger genes like *AS1/2*, *BOP1/2*, *SAW1/2* to inhibit the expression of *KNOX I* (*STM* and *BP*). During the development of leaf margin serration, KNOX I genes (*STM*, *KNAT2* and *KNAT6*) positively regulate the serration degree. *JLO* induces and activates the expression of *STM* and *BP*. ATH1 forms heterodimer with STM and BP.While *CUC2* can significantly inhibit the expression of *KNAT2* and *KNAT6* genes. JAG promotes the expression of *JLO* (the activating gene of KNOX I), and inhibits the expression of CUC2 (the inhibitor of KNOX I) thereby promoting the expression of KNOX I through two-way regulation. *KNOX I* (*STM* and *BP*) further promotes the expression of cytokinin gene *IPT7* and inhibits the expression of gibberellin synthesis gene *GA20ox1*, thereby regulating the hormone level at the leaf margin and finally establishing the leaf serration morphology. The solid arrows indicate results that have been experimentally determined, whereas dashed arrows indicate speculated effects supported by the literature.

Gibberellin and brassinolide control leaf growth by promoting cell proliferation and expansion [43,44]. *PIN1* is also required for organ initiation, boundary restriction, and growth [45]. *ERECTA* (*ER*) can control cell proliferation by inhibiting auxin expression [46]. In *LtuJAG* overexpression lines, the expression of *PIN1* and *ER* was not significantly different from that of the wild type, indicating that *JAG* may not be directly related to auxin biosynthesis. As a downstream target gene of *KNOX I*, *IPT7* in *A. thaliana* was induced to be expressed by *STM* during apical meristem differentiation [47]. *STM* also inhibited gibberellin activity by reducing the level of *GA20ox1*. In addition, the expression of *GA20ox1* in tomato was negatively correlated with the degree of leaf margin serration, whereas *IPT7* was positively correlated with it [48]. *LtuJAG* significantly upregulated the expression of *IPT7* and decreased the expression of *GA20ox1*, indicating that *JAG* can also participate in the antagonism between hormones, thereby contributing to the differentiation of meristem organs. Whether it regulates hormones through the *KNOX I* pathway requires further research. In conclusion, we speculate that the boundary limiting factors *JLO* and *ATH1*, the upstream activators of the *KNOX I* gene, were directly activated by *JAG*, resulting in leaf primordia with disorganized polarity that sometimes fail to complete morphological development(Figure 8), but further studies are needed to determine the validity of this hypothesis. The mechanism by which genes controlling leaf morphogenesis interact at different developmental stages is unclear. Investigating this will be a focus of our future research.

## 4. Materials and Methods

### 4.1. Plant Materials and Growth Conditions

We found that the number of leaf serration increased with time in *L. tulipifera* and ended at senescence. Moreover, in a previous study, we had determined the timing and location of leaf initiation, leaf polarity establishment, leaf serration formation and leaf morphogenesis in *L.chinense* and *L. tulipifera* using SEM and paraffin section observation [49]. Our focus was on whether *LtuJAG* genes were involved in the process of altering the number of leaf serrations. Therefore, *L. tulipifera* trees were studied, originating from South Carolina, USA, were planted in a provenance trial plantation in the Xiashu Forest Farm in Jurong County, Jiangsu Province, China (119°13′20″ E, 32°7′8″ N) in 1993 [50].

**Figure 9:**
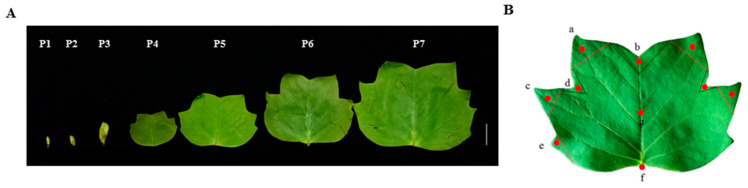
Schematic diagram of sampling in *L. tulipifera*. (**A**) Different stages of leaf development in *L. tulipifera*. P1–P2: leaf bud growth stage; P3: young leaf stage; P4–P6: mature stage; P7: senescence stage; (**B**) Different parts of leaf in *L. tulipifera*, a, c, and e: leaf tooth tip, b and d: leaf tooth sinus, g: middle of leaf, f: petiole.

To investigate the expression pattern of *LtuJAG* in different tissues, leaves, stems, stamens, pistils, flower buds, sepals, leaf buds, and petals of *L. tulipifera* were collected in April 2018. To verify the time-specific expression pattern, leaves at different stages of development (leaf bud germination, young leaves, mature leaves, and senescence) were collected from March to August 2018 (Figure 9A). As our focus lies in studying the leaf polarity establishment, leaf serration formation and leaf morphogenesis, we collected leaf buds and leaf from the corresponding stages, namely leaf bud germination stage (P1 and P2), young leaf stage (P3), leaves with three lobes, five lobes and six lobes, divided into mature stage (P4 and P6) and senescence stage(P7). Current studies emphasized that the generation of leaf margin serrations requires hormonal convergence at the leaf tooth tip, while boundary genes are confined at the leaf tooth sinus [45]. To explore the effect of *JAG* genes as boundary suppressors on different parts of leaf in *L. tulipifera*, leaves were categorized into the protruding parts of the leaf margin (a, c, and e), concave parts of the leaf margin (b and d), petiole (f), and the middle part of the leaf (g) (Figure 9B), three replicates from each of the seven samples (approximately 1 g each) were separated using sterilized scissors. All samples were frozen immediately in liquid nitrogen and stored at −80 °C for RNA extraction, subsequent molecular cloning and real-time quantitative PCR.

Transgenic and wild-type plants were grown in a Columbia-0 (Col-0) background with *Nicotiana benthamiana*, and planted in a 6:3:1 mixture of potting soil: vermiculite: perlite in a 23 °C illumination incubators under long days (16 h light/8 h dark, 80% humidity).

### 4.2. Rapid Amplification of cDNA Ends (RACE), Sequencing, and Sequence Analysis of LtuJAG

The *JAG* EST number (gnl|Liriodendron|b4_c58305) was obtained from the transcriptome database of *L. tulipifera* (http://jlmwiki.plantbio.uga.edu/aagp/) (accessed on 24 July 2018). RACE cloned primers were designed using Oligo 7 software (Table S1). RACE was conducted using a SMARTer^®^RACE 5/3 kit (Takara Biomedical Technology, Beijing, China) according to the manufacturer’s instructions for amplifying full-length *LtuJAG*, and the amplified PCR product was then ligated into the pEASY vector using a pEASY-Blunt Zero cloning kit (Transgen Biotech, Beijing, China) and verified by sequencing (GenScript Biomedical Technology, Nanjing, China).

*LtuJAG* Open Reading Frames (ORFs) were predicted using the NCBI ORF finder. The physicochemical characteristics of *LtuJAG* were analyzed using Expasy Protparam (https://web.expasy.org/protparam/) (accessed on 16 October 2021). Multiple alignments of *JAG* amino acid sequences from various plant species were edited using the DNAMAN 8.0 software. A phylogenetic tree of the aligned sequences was created with MEGA 5.1 software using the neighbor-joining method based on 1000 bootstrap replicates [51]. The promoter was analyzed using the online software PlantCARE to predict putative cis-elements.

### 4.3. Subcellular Localization

To investigate localization where *LtuJAG* functions in the leaf cell, its subcellular localization was performed using the *Agrobacterium* injection method as described earlier by Lin et al. [52]. Briefly, the CDS of *LtuJAG* (without a stop codon) was inserted into the pBI121 vector containing the 35S promoter with an eGFP protein, according to the CloneExpress^®^Ultra One Step Cloning Kit instructions (Vazyme Biotech, Nanjing, China). Next, the constructed 35S::LtuJAG-GFP and the positive control 35S::eGFP plasmids were inserted into Agrobacterium GV3101, which was then injected into the *N. benthamiana* leaves [53]. To monitor the location of *LtuJAG* more precisely, the nucleus-specific dye DAPI was utilized after transfection at 22 °C for 24 h in the dark. We observed the green fluorescent protein (eGFP) in the tobacco leaves at 2 days after transfection using a Zeiss LSM 710 confocal microscope (Carl Zeiss AG, Jena, Germany). Green fluorescence signals may appear in the cell membrane, nucleus, and cytoplasm. DAPI is a blue fluorescence signal that represents the nucleus location.

### 4.4. Agrobacterium-Mediated Transformation

To generate the *LtuJAG*—overexpressing transgenic *A. thaliana*, the *LtuJAG* coding region was cloned into an improved pBI121-GUS vector downstream of the 35S promoter through the XbaI and BamHI sites to generate the plasmid 35S::pBI121-LtuJAG. This plasmid was inserted into *Agrobacterium* GV3101, which was transferred to *A. thaliana* (Col-0) using the floral-dip method [54]. The T_0_ seeds of the transgenic plants were screened using a 1/2 MS solid medium containing 50 mg/L kanamycin. The positive seedlings could grow normally about 10 days after seed germination—also observed in transgenic lines of the T_1_ generation. The selection of homozygous transgenic lines through T_2_ and T_3_ generations was performed as described by Mahmood et al. [55]. To monitor the transcript levels of transgene in *LtuJAG* transgenic lines, total RNA was isolated from the rosette leaves of WT (Col-0) and *LtuJAG* transgenic plants at 28 days after seed germination using a DP441-RNAprep Pure Plant Kit (Tiangen, Beijing, China) according to the manufacturer’s instructions. Primer pairs, LtuJAG-qRT-F and LtuJAG-qRT-R, were used in quantitative RT-PCR. *AtACTIN2* was used as an internal control to normalize the expression values of the transgene transcript level [56]. The used primers were listed in Table S1. The relative expression was calculated using the 2^−∆∆Ct^ method [57]. Three biological replicates and three technical replicates. Data were analyzed statistically using the one-way ANOVA Tukey’s test.

### 4.5. GUS Histochemical Assay and Phenotypic Characterization

It is known that JAG is a class of transcription factor that is specifically expressed in the apical meristem. To investigate *LtuJAG* promoter expression pattern during differentiation of the apical meristem, we cloned promoter sequences. About 2Kb bp fragments upstream of the translational region of the *LtuJAG* gene were inserted into the plasmid pBI121-GUS at the corresponding restriction sites in place of the 35S promoter region, thereby forming a pBI121-pLtuJAG::GUS construct and transformed into *A. thaliana*. T_2_ generation transgenic positive lines were used as research material after screening and identification, these seeds and seedlings at the two-leaf, four-leaf, and six-leaf stages were collected, respectively, at 0, 2, 4, 6, 8, 12 days after seed germination. To understand the tissue-expression pattern, we collected leaves, flowers, stems and siliques from the reproductive stage at 42 days after seed germination, of which GUS activity was detected using GUS histochemical analysis, specific method as previously described by Jefferson et al. [58]. First, tissue samples (1 g each) were submerged in 10 mL X-gluc solution (0.1% 5-bromo-4-chloro-3-indolyl-beta-D-glucuronic acid (X-gluc), 1% dimethyl formamide, 50 mM sodium phosphate [pH 7.0]) at 37 °C for 24 h. The treated tissues were then decolorized in 75–100% ethanol. The samples were photographed using a Leica DM500 microscope (Shanghai, China).

To explore the effect of the *LtuJAG* gene on leaf morphology, the 5–12th rosette leaves (except 1–4th cotyledons) of the T_3_ generation homozygous WT and *LtuJAG* overexpression lines grown for 32 days, were photographed using a Fujifilm Finepix S5100 digital camera (Tokyo, Japan), and the JPEG images were used to calculate the lamina length, lamina width, petiole length, lamina area of 12 replicated individual lines using Digimizer (www.digimizer.com) (accessed on 12 November 2021) software, the specific method referring to Luo et al. [59]. We aimed at studying the leaf size differences between the wild-type and overexpression strains and therefore data were analyzed statistically using Student’s *t*-test to pairwise comparison, and differences were considered significant when values *p* < 0.01 and *p* < 0.05.

### 4.6. Real-Time Quantitative PCR

To study the temporal expression pattern of genes associated with leaf serration, we examined the transcript levels of leaf serration related genes in wild-type *A. thaliana* leaves at different growth stages and confirmed all genes were specifically expressed at leaf primordia differentiation (10 days after seed germination), and to some extent, some genes were not even expressed at the maturation (25 days after seed germination), reproductive (40 days after seed germination), and senescent stages (50 days after seed germination). To explore the effect of *LtuJAG* on leaf serration related genes, triplicate seedling samples of the homozygous WT and *LtuJAG* overexpression lines (with the lobed leaf phenotype) at the initial leaf primordia stage (10 days after seed germination), were frozen in liquid nitrogen and stored at −80 °C in preparation for gene expression testing using real-time quantitative PCR.

Total RNA was extracted from different samples of *A. thaliana* and *L. tulipifera* using a total RNA extraction kit (Tiangen, Beijing, China). cDNA was synthesized using the PrimeScript II 1st strand cDNA Synthesis Kit (TaKaRa Biomedical Technology). Quantitative real-time PCR was performed in triplicate using a SYBR Premix Ex Taq kit (TaKaRa Biomedical Technology) on an Applied Biosystems real-time PCR system. *AtACTIN2* of *A. thaliana* and *LcActin97* of *Liriodendron chinense* were used as internal controls with the primers listed in Table S1 [56,60]. For PCR, initial denaturation was performed at 95 °C for 60 s, which was followed by 40 cycles of 5 s at 95 °C and 34 s at 60 °C [61]. Three biological replicates and three technical replicates. The relative expression was calculated using the 2^−∆∆Ct^ method [56]. Data were analyzed statistically using the one-way ANOVA Tukey’s test.

## 5. Conclusions

In this study, we cloned and characterized the *LtuJAG* gene involved in the development of leaf polarity in *L. tulipifera*. This gene is a nuclear localized transcription inhibitor that is highly expressed in the lateral organs. We speculate that *LtuJAG* activates the boundary limiting factors *JLO* and *ATH1* and upregulates the negative regulator of the KNOX I, thereby causing a downregulation of the KNOX I gene. This may disrupt the balance of endogenous hormones, affecting the development of leaf primordia and producing defective leaves. Our study provides evidence that *JAG* plays a role in the development of leaf polarity.

## Data Availability

Not applicable.

## Supplementary Materials

The following supporting information can be downloaded at: https://www.mdpi.com/article/10.3390/ijms23031322/s1.
